# Stabilized and unstabilized sampling methods result in differential fecal 16S rRNA microbial sequencing results

**DOI:** 10.1371/journal.pone.0324351

**Published:** 2025-08-13

**Authors:** Christopher E. Stamper, Andrew J. Hoisington, Joseph C. Ellis, Christopher A. Lowry, Lisa A. Brenner

**Affiliations:** 1 Veterans Health Administration, Rocky Mountain Mental Illness Research Education and Clinical Center (MIRECC) for Veteran Suicide Prevention, Rocky Mountain Regional Veterans Affairs Medical Center (RMRVAMC), Aurora, Colorado, United States of America; 2 Department of Physical Medicine and Rehabilitation, University of Colorado Anschutz Medical Campus, Aurora, Colorado, United States of America; 3 Military and Veteran Microbiome Consortium for Research and Education (MVM-CoRE), Aurora, Colorado, United States of America; 4 Department of Systems Engineering and Management, Air Force Institute of Technology, Wright-Patterson Air Force Base, Dayton, Ohio, United States of America; 5 Netellis LLC, Knoxville, Tennessee, United States of America; 6 Department of Integrative Physiology, Center for Neuroscience, and Center for Microbial Exploration, University of Colorado Boulder, Boulder, Colorado, United States of America; 7 Veterans Health Administration, Eastern Colorado Health Care System, Rocky Mountain Regional Veterans Affairs Medical Center (RMRVAMC), Aurora, Colorado, United States of America; 8 Department of Psychiatry, University of Colorado Anschutz Medical Campus, Aurora, Colorado, United States of America; 9 Department of Neurology, University of Colorado Anschutz Medical Campus, Aurora, Colorado, United States of America; City University of New York, UNITED STATES OF AMERICA

## Abstract

Over the past decade, studies have been conducted to increase the understanding of associations between the fecal microbiome and human health. In conjunction, researchers have investigated the effects of study design, methods, molecular processing, and sequencing techniques. However, a lack of standardization of fecal sample collection methodology has introduced heterogeneity in sequencing results. Sources of variability include sample collection methods, storage temperatures, and transport times. Here we present 16S rRNA gene amplicon sequencing results from two sample collection methods (unstabilized sterile swab and stabilized OmniGene Gut Kits) collected from the same fecal specimens. The paired samples were collected either at the research facility or the participants’ home and ground shipped to the research facility at ambient temperature. Therefore, samples were exposed to variable temperatures and transport times. We found that fecal sample collection methods resulted in taxonomic and diversity differences that showed distinct patterns between swab and OmniGene samples. Swab samples were disproportionally affected by increased transport time, but differences in taxa and diversity were driven more by sample collection method, as compared to transport time. Based on previous studies, many of the taxa that were associated with sample collection methods and transport times have clinical relevance. Collectively, this research highlights: 1) the need for further standardization of methods for fecal microbiome studies; 2) limitations of direct comparisons between different fecal sample collection methods; and 3) the importance of careful consideration of sample collection methods for future studies and meta-analyses.

## Introduction

Over the past decade there has been increased focus on associations between the fecal microbiome and disease states [1,2], and the utility of microbiome-based interventions to improve human health [3,4]. During this period of rapid expansion of the field, methodological and statistical best practices were not yet fully established or vetted [5,6]. Indeed, methodological best practices are still being improved on in the areas of sample collection [7,8], sample storage [9], DNA extraction [10], and polymerase chain reaction (PCR) primers [11].

Sample collection and processing methods, including time from collection to freezing (transport time), impact the quality and reliability of microbiome data [12–16]. A temperature and time dependent association between microbial communities and shipping of unstabilized fecal samples [17,18] led to the practice of fresh freezing samples, when possible, and the introduction of stabilizing reagents when frozen shipping conditions were not feasible. Although most stabilizing reagents are effective in preventing dramatic microbial community changes during shipping, different stabilizing reagents recover different microbial communities from the same fecal sample [12–14]. Despite the apparent advantages of stabilizing reagents or immediately freezing samples, standardization of sample collection practices has not occurred, resulting in challenges comparing results across studies and combining sequence data for meta-analyses.

In the present study, we collected swab and OmniGene samples from the same fecal specimen provided by participants in three clinical studies conducted over four years. Participants provided their swab and OmniGene samples either at the research facility (i.e., frozen shortly after collection) or at home (i.e., ground shipped back to the research facility at ambient temperature). The aims of this study were to determine: 1) if there were microbial taxonomic and community differences between paired swab and OmniGene samples (*n* = 325 each); 2) if transport time affected microbial taxa and communities of swab and OmniGene samples (*n* = 325 each); and 3) if swab samples collected at the research facility (non-shipped), had taxonomic profiles and microbial communities more similar to their paired OmniGene samples (*n* = 104 each). We hypothesized that: 1) there would be differences in microbial taxa and communities between sample collection methods; 2) transport times would affect microbial taxa and communities from swab but not OmniGene samples; and 3) non-shipped swabs would be more similar in terms of microbial taxa and communities to their paired OmniGene samples.

## Materials and methods

### Samples

All samples in the present study were from the Military and Veterans Microbiome Consortium for Research and Education (MVM-CoRE). Participant samples were from three studies to include: 1) a survey of United States (US) Veterans (*n* = 181; Colorado Multiple Institutional Review Board [COMIRB] Protocol # 15–1885, participants recruited April 1, 2016 – present); 2) a study of US Veterans with a history of traumatic brain injury (*n* = 43, COMIRB Protocol # 18–2820, participants recruited July 1, 2019 – June 30, 2022); and 3) a study of non-Veterans seeking Emergency Department services following a recent mild traumatic brain injury (*n* = 101; COMIRB Protocol # 19–1307, participants recruited December 1, 2019 – June 30, 2021). All procedures performed were in accordance with the ethical standards of COMIRB, and with the1964 Helsinki Declaration and its later amendments or comparable ethical standards. Informed consent was obtained from all participants included. More information to characterize the sample is available elsewhere [19–21]. Specifically, participants with two fecal microbiome samples from the same fecal specimen were selected with: 1) a dry sterile dual tipped BD BBL™ CultureSwab™ EZ II swab (Cat. No. B220144, Fisher Scientific, Pittsburgh, PA, USA) using the “first wipe” method without stabilizing reagents as previously reported by Brenner et al., [22]; and 2) an OmniGene Gut Kit (Cat. No. OMR-200, DNA Genotek, Ottawa, Canada) using manufacture provided instructions. Samples were either provided in-person during a study visit and were frozen shortly after collection, or collected at the participant’s residence and shipped to the research facility. Sample shipment was by standard ground shipping at ambient temperature in a padded envelope without an ice pack. Upon arrival at the research facility, shipped swabs were placed directly into the freezer (–80 °C). OmniGene samples were aliquoted into working volumes prior to being placed in the freezer (–80 °C). Aliquoting of OmniGene samples was completed within days of the sample arrival. Transport time (days) for both OmniGene and swab samples was calculated as the difference between the day the samples were reported being collected and the time of freezing at the laboratory. Some participants provided swab and OmniGene samples in person at the research facility (*N* = 104), which were frozen shortly after collection. Using this information, a dichotomous variable was created for shipping status as either “shipped” or “not-shipped”.

### Sample processing

DNA was extracted from microbiome samples using the PowerSoil DNA extraction kit (Cat. No. 12955−4, Qiagen, Valencia, California, USA) and DNA was quantified via Quant-IT dsDNA Assay Kit, conducted in triplicate (Cat. No. Q33120, Invitrogen, Waltham, MA, USA). Marker genes in isolated DNA were polymerase chain reaction (PCR)-amplified using GoTaq Master Mix (Cat. No. M5133, Promega, Madison, WI, USA) and 515 F (5′-GTGCCAGCMGCCGCGGTAA-3′), 806 R (5′-GGACTACHVGGGTWTCTAAT-3′) primer pair (Integrated DNA Technologies, Coralville, IA, USA) targeting the V4 hypervariable region of the 16S rRNA gene modified with a unique 12-base sequence identifier for each sample and the Illumina adapter as previously described in Caporaso and Lauber [23]. The thermal cycling program consisted of an initial step at 94 °C for 3 minutes followed by 35 cycles (94 °C for 45 seconds, 55 °C for 1 minute, and 72 °C for 1.5 minutes), and a final extension at 72 °C for 10 minutes. PCRs were run in duplicate and the products were visualized on an agarose gel to ensure successful amplification prior to pooling. PCR products were cleaned, normalized (Cat. No. 28104, Qiagen) and sequenced by the University of Colorado Anschutz Center for Microbiome Excellence. Sequencing was performed on an Illumina MiSeq using V2 chemistry and 300 cycle, 1 × 250-bp sequencing. Demultiplexed single-end sequences were deposited in the NCBI Sequence Read Archive (BioProject accession ID: PRJNA1101562).

### Sequence processing

Sequencing data were processed and analyzed using the Quantitative Insights Into Microbial Ecology program (QIIME2 v. 2023.5) [24] and the open-source statistical software R v. 4.2.2 [25] (https://www.R-project.org). Only forward read sequences were used in the previously established Earth Microbiome Project [26] single-end sequence processing pipeline. The samples included in this analyses were sequenced in 14 different runs. Each sequencing run was processed individually, and samples included in this analysis were subset from their run feature table and merged together prior to rarefaction and core diversity analysis. The Deblur algorithm [27] was used to denoise demultiplexed sequences and filtered based on quality metrics previously described in Bokulich et al. [28]. Quality-filtered sequences were assigned taxonomic classification with the minimum confidence threshold set to 70% based on the Silva database (v. 138) [29] and rarefied to 10,000 sequences per sample prior to downstream analysis. The rarefaction curve (S1 Fig) was referenced when determining the rarefaction level. The alpha diversity metrics used to determine within-sample diversity were observed features (also referred to as species richness) and Shannon diversity. The beta diversity metrics to determine between-sample diversity were weighted and unweighted UniFrac [30].

### Data analysis

Statistical tests were conducted with a two-tailed alpha level of 0.05 and when appropriate corrections due to multiple comparisons were made using the Bonferroni method. For alpha diversity, linear mixed effects (LME) modelling was used to examine differences between fixed effects of sample collection methods, transport time, and/or the interaction of sample collection method and transport time with the model accounting for repeated measures as a random effect due to the paired sample study design. Spearman correlation was used to examine the association of alpha diversity metrics with transport time by sample collection method. For beta diversity metrics, PERMANOVA within the vegan package in R from Adonis2 command was used to examine sample collection method, transport time, and the interaction of sample collection method and transport time (999 permutations, strata = participant to account for the repeated measures of the paired sample study design) [31]. Biplots of swab samples at the phylum and class level for weighted UniFrac for the transport time variable were performed using constrained correspondence analysis (CCA) within the vegan package in R [31]. Taxonomic analysis was conducted at the phyla, class, and genera levels. Differential abundance was assessed for sample collection method and transport time using ANCOMBC2 package in R (prevalence filter set to 0.30 and Bonferroni correction used due to multiple comparisons) [32]. For taxa determined to be differentially abundant by sample collection method or transport time by ANCOMBC2, Spearman’s correlation was used to examine the association of relative abundance with transport time by sample collection method. This manuscript was developed to meet the STORMS reporting checklist, v1.03 [33].

## Results

This study included 650 paired swab and OmniGene samples (*n* = 325 each). There were 4,060 unique observed features across all samples. Transport time ranged from zero (non-shipped) to ten days (3.4 ± 2.7 days [mean ± standard deviation]).

### Diversity and bacterial community structure differences between Swab and OmniGene samples

**Fig 1:**
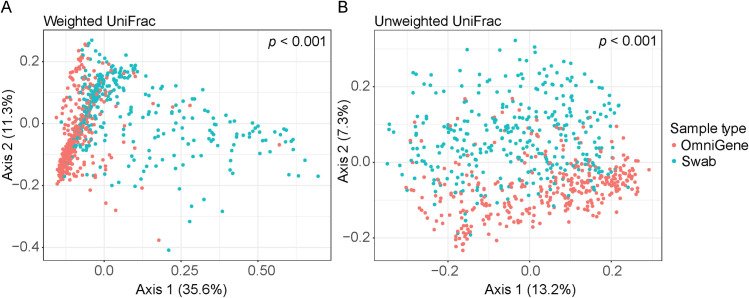
PCoA plots illustrating differences in microbial community structure between the sample collection methods. A) Weighted UniFrac distance metric. B) Unweighted UniFrac distance metric. *p*-values from PERMANOVA.

Differences between paired swab and OmniGene samples were found in one alpha diversity and both beta diversity measures. For alpha diversity, swabs had lower observed features 160.1 ± 55.6 than OmniGene samples 163.7 ± 53.9 (LME; *p *= 0.003). In contrast, analysis using Shannon diversity, a measure of both richness and evenness, revealed there was no difference between swab 4.7 ± 1.0 and OmniGene samples 5.1 ± 0.75 (LME; sample collection method, *p *= 0.80). For beta diversity, swab and OmniGene samples had different microbial community structures in weighted and unweighted UniFrac metrics (both PERMANOVA, *p *< 0.001; Fig 1).

**Fig 2:**
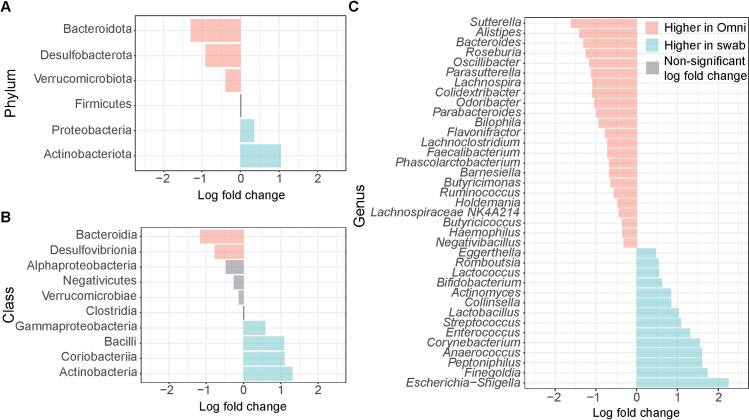
Log fold change at the phylum (A), class (B), and genus (C) level. Log fold change values were relative to the relative abundance of taxa for swabs, therefore positive values indicate higher abundance in swabs and negative values indicate higher abundance in OmniGene. Statistically significant (ANCOMBC2; Bonferroni corrected, *p* < 0.05) differential abundance is indicated by a colored bar and gray bars indicate no statistical significance. A table with statistics can be found in the supplemental material (S1 Table).

There were numerous taxa that were differentially abundant between swab and OmniGene samples (Fig 2). Specifically, there were five phyla, six classes, and thirty-seven genera that were differentially abundant between the sample types (ANCOMBC2; S1 Table).

### Impact of transport time on swab and OmniGene microbial communities

**Fig 3:**
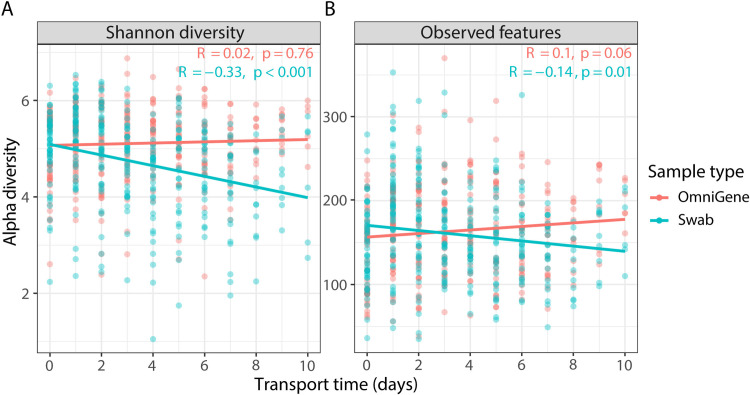
Spearman correlation of alpha diversity metrics by sample collection method. A) Shannon diversity. B) Observed features. Rho (*R*) and *p*-values from Spearman correlation.

The impact of increasing transport time on microbial communities differed between swab and OmniGene samples. OmniGene samples were stable based on alpha diversity: Shannon diversity (Spearman; *R* = .02, *p *= 0.76; Fig 3A) and observed features (Spearman; *R* = 0.1, *p *= 0.06; Fig 3B). Similarly, beta diversity (weighted UniFrac; PERMANOVA, *p *= 0.37) and the relative abundances of specific taxa were stable in OmniGene samples (ANCOMBC2; no significant association between the relative abundance of specific taxa at phylum, class, or genus level and transport time).

**Fig 4:**
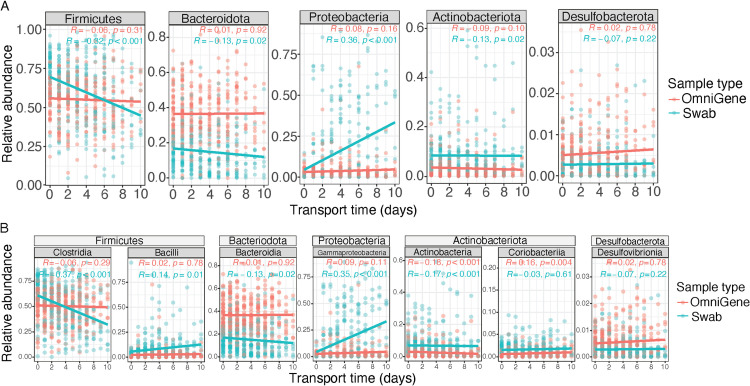
Associations between relative abundances of specific taxa with transport time by sample collection method at the A) phylum- and B) class-level. Scatter plots with line of best fit over transport time colored by sample collection method. Rho (*R*) and *p*-values from Spearman correlation.

However, the diversity and microbial community structure of fecal microbiomes collected using swabs were not stable as both alpha diversity metrics were negatively correlated with transport time: Shannon diversity (Spearman; *R* = –0.33, *p* < 0.001; Fig 3A) and observed features (Spearman; *R* = –0.14, *p *= 0.01; Fig 3B). Similarly, the microbial community structure of swab samples shifted over transport time based on analysis using weighted and unweighted UniFrac (both PERMANOVA; *p *< 0.001). There were changes in the relative abundances of specific taxa based on transport time at the phylum (Fig 4A), class (Fig 4B), and genus (S2 Fig) level. There was a notable influence of transport time on Proteobacteria and the related class Gammaproteobacteria based on CCA results (S3 Fig).

### Differences in bacterial community structure between non-shipped paired swab and OmniGene samples

**Fig 5:**
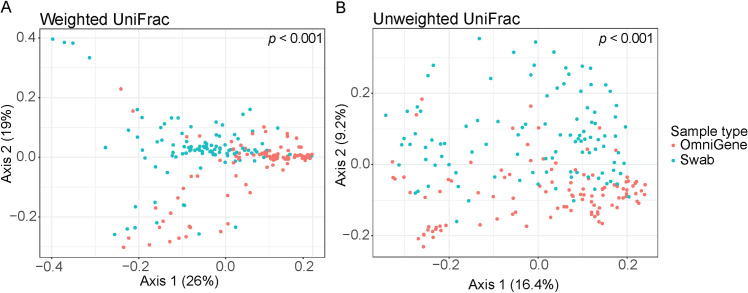
Beta diversity PCoA plot of the subset of non-shipped paired swab and OmniGene samples. A) Weighted UniFrac distance metric. B) Unweighted UniFrac distance metric.

We examined a subset of non-shipped paired swab and OmniGene samples (*n* = 104 each) to directly compare the impact of sampling methods, absent of transport time. In this subset, differences in bacterial community structure between non-shipped paired swab and OmniGene samples were found in one alpha diversity and both beta diversity measures. For alpha diversity, swabs had lower observed features 156.4 ± 50.5 than OmniGene samples 169.0 ± 62.6 (LME; *p *= 0.01). In contrast, analysis of Shannon diversity, a measure of both richness and evenness, revealed that there was no difference between non-shipped paired swab 5.1 ± 7.2 and OmniGene samples 5.1 ± 0.85 (LME; *p *= 0.91). For beta diversity, there were differences in microbial community structure between the non-shipped paired swab and OmniGene samples based on analysis using weighted and unweighted UniFrac measures (both PERMANOVA, *p *< 0.001; Fig 5).

**Fig 6:**
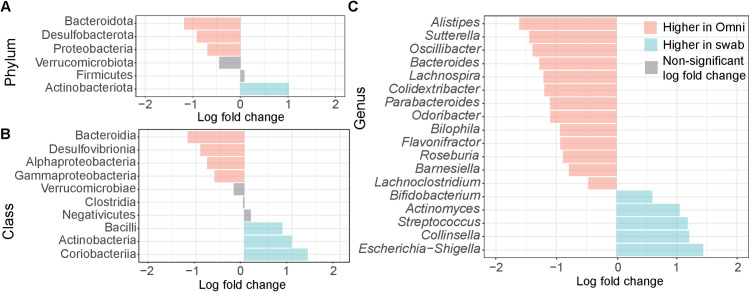
Log fold change of phylum (A), class (B), and genus (C) level in the subset of non-shipped paired swab and OmniGene samples. Log fold change values were relative to the relative abundance of swabs, therefore positive values indicate higher relative abundance in swabs and negative values indicate higher relative abundance in OmniGene. Statistically significant (ANCOMBC2; Bonferroni corrected, *p* < 0.05) differential abundance is indicated by a colored bar and gray bars indicate no statistical significance. A table with statistics can be found in the supplemental material (S2 Table).

There were numerous taxa that were differentially abundant at the phylum, class, and genus level between swab and OmniGene samples (Fig 6). Specifically, there were four phyla, seven classes, and eighteen genera that were differentially abundant between swab and OmniGene samples.

The differentially abundant taxa at the phylum, class, and genus level between swab and OmniGene samples were nearly all consistent between the non-shipped and the full dataset, indicating a strong impact of sample collection method on taxonomic variation (Figs 2 vs 6). Interestingly, the phylum Proteobacteria and the related class Gammaproteobacteria both changed from swab samples, having the higher relative abundance in the full dataset, to OmniGene having higher relative abundance in the non-shipped subset. This is in concordance with the result of Proteobacteria, Gammaproteobacteria, and the related genus *Escherichia-Shigella* showing an increase in relative abundance in swab samples with increased transport time.

## Discussion

In the present study, we investigated differences in fecal collection methods between swab and OmniGene samples, and the impact of transport times on taxa and diversity of microbial communities of the fecal microbiome. This study provides data from samples that are identical other than the collection method. Key findings from this study were: 1) fecal sample collection methods resulted in taxonomic and diversity differences that showed distinct patterns; 2) swab samples were disproportionally impacted by increased transport times; 3) differences in taxa and microbial community structure were driven more by sample collection methods than transport times; and 4) many of the taxa that were associated with sample collection methods and transport times have been shown to have clinical relevance.

Gram-negative bacteria were overrepresented in OmniGene samples including Bacteroidota and Proteobacteria (at the phylum level), as well as *Sutterella* and *Bacteroides* (at the genus level). It has been reported that OmniGene kits recover higher abundances of gram-negative bacteria relative to fresh frozen samples [13,14]. Gram-negative bacteria have a cell membrane and peptidoglycan layer structure that is thinner than gram-positive bacteria, resulting in increased cell lysis and subsequent DNA exposure [14]. Stabilizing reagents in OmniGene collection kits are known to include lysing agents [34], which may explain the overrepresentation of gram-negative bacteria [13,35]. Due to the compositional nature of relative abundance, overrepresentation of gram-negative bacteria may also account for underrepresentation of gram-positive bacteria [5]. Indeed, for gram-positive bacteria, we observed lower Actinobacteriota at the phylum level as well as *Collinsella* and *Bifidobacterium* at the genus level in OmniGene samples relative to swabs, which has also been observed previously [14,36–38].

Increased transport time impacted microbial communities in swab samples, but not in OmniGene samples. Previous studies have reported that multiple stabilizing reagents are effective at preventing increases in microbial dissimilarity over various periods of time [13,14,39–41]. Unstabilized fecal swab samples have been used in numerous studies, with consistent findings regarding the selective overgrowth in facultative anaerobes, when samples are not frozen shortly after collection. Roesch et al. reported that 72 hours at ambient temperature was associated with an ~ 10% change in community composition [17]. Researchers from the American Gut Project highlighted the blooming of facultative anaerobes in swabs (i.e., mainly taxa in the classes of Gammaproteobacteria and Bacilli) and recommended culling of select ASVs to alleviate the issue [15]. In addition to blooming of specific taxa, we observed negative relationships between transport time and other taxa in swab samples. For example, Clostridia and the related genus *Faecalibacterium* showed a negative relationship with transport time in the present study and previous studies [17]. However, the biological mechanism behind this negative relationship is unclear, though it may simply be an artifact of the compositional nature of 16S rRNA gene amplicon sequencing [5].

In the absence of transport times (i.e., non-shipped samples), significant differences in taxa and diversity between swab and OmniGene samples persisted. In fact, nearly all the taxonomic differences between the sample collection methods in the non-shipped subset were shared with the full dataset. Suggesting that sample collection methods have more influence on microbial taxa and diversity than transport times. One interesting observation when comparing the non-shipped subset and the full dataset was related to two notable changes in taxonomic abundances. Proteobacteria and Gammaproteobacteria showed higher relative abundance in non-shipped OmniGene samples relative to non-shipped swab samples. However, if transport time was considered, Proteobacteria and Gammaproteobacteria showed higher relative abundance in swab samples. Other researchers have also noted stabilizing reagents are associated with an increase in abundance for both taxa [12,34,38], and a positive relationship with transport time in unstabilized samples [15,18,36]. Additionally, the nature of these relationships is supported by the aforementioned overrepresentation of gram-negative bacteria and blooming of facilitative anaerobes hypotheses.

In this study, we observed that many of the taxa affected by sample collection methods and transport times have clinical relevance [4,19,42]. The Firmicutes/Bacteroidetes ratio, a global measure of health previously used by several studies [43–47], is impacted by both stabilizing reagents (i.e., Bacteriodota was higher in OmniGene samples) and transport time of unstabilized samples (i.e., Firmicutes negatively correlated with transport time). Several facultative anaerobes shown to increase in swabs with longer transport time have also been associated with irritable bowel syndrome/ inflammatory bowel disease [48,49]. For example, seven genera (*Escherichia, Bacteroides, Bifidobacterium, Rumminococcus, Faecalibacterium, Roseburia,* and *Dorea*) from the present and previous studies [12,15,18] were associated with increased transport time for unstabilized samples have also been linked to the gut microbiome of participants with IBS or IBD [50–52]. In relation to the gut-brain axis, increased abundance of Actinobacteria [53,54], Coriobacteriia [53,55], and Verrucomicrobiota [54] have been associated with positive outcomes in studies of posttraumatic stress disorder and depressive-like behaviors. Yet, these three taxa were also shown to be associated with sample collection methods in the present study. These collective co-occurring associations are not inconsequential and as noted by Maghini et al., the lack of standardization of sample collection and processing methods may pose a ‘reproducibility crisis’ in reference to irreproducible health-related microbial signatures [13].

There are increased efforts to promote standardization of practices. Collaborating centers that focus on standardization and quality control (i.e., International Human Microbiome Standards Project [9,56] and Microbiome Quality Control Project [57]), and implementation of tools to promote rigor (e.g., Strengthening the Organization and Reporting of Microbiome Studies [STORMS] checklist [33]) represent important steps forward. The continued movement toward methodological standardization and emerging technologies are expected to positively impact future research. In addiiton, more attention may need to be directed toward meta-analyses and the methods associated with the studies being compiled. For example, a recent meta-analyses compiled sequencing data from sixteen different studies that used eleven different sample collection methods [58] and another compiled sequencing data from 67 studies from 2012–2023 [59] The later was conducted during a period of rapid development for the field, nonetheless the authors made no mention of sample collection methods.

Bioinformatic tools could assist in post-sequencing correction to remove known methodological bias. Amir et al. presented one bioinformatic process for correcting the effects of transport time at ambient temperature on unstabilized samples by culling systematically identified ASVs known to artificially bloom [15]. Albeit a relatively blunt approach, it was validated in a large dataset, and shown to restore known microbiome associations with age, race, and sex in American Gut Project data. Yet, this approach to remove potentially abundant and health relevant ASVs has not been widely adopted in the field. The convergence of large amounts of publicly available microbiome data and high-power computing techniques present opportunities for new tools and mechanisms to correct for methodological bias. Validated tools have the potential to improve the reliability and reproducibility of microbiome research.

This study has limitations including that approximately one-third of samples had longer than average transport times (average transport time is three to four days). Samples with transport time over 4 days, experienced unknown climatic conditions, which is pertinent, as this study took place in the Denver metro area of Colorado, with variable climatic conditions throughout the year. Additionally, the transport time data were not normally distributed and exhibited a positive skew. In response we employed Spearman correlation to accommodate for this and included both the Rho (*R*) and *p* values for transparency when interpreting these relationships. Additionally, it should be recognized that there are financial and other burdens associated with sampling with OmniGene kits that may be prohibitive to using this sampling method, particularly to researchers with limited resources or working in remote areas. A strength of this study was that all the samples were paired with both a swab sample and an OmniGene sample from the same fecal specimen.

In conclusion, we examined the impact of two commonly used sample collection methods and the effects of transport time on microbial taxa and diversity based on 16S rRNA gene amplicon sequencing data. We determined that OmniGene kit samples were characterized by overrepresentation of gram-negative bacteria. Fecal swab samples were characterized by increases in the relative abundance of facultative anaerobes with transport time. The differences in microbial taxa and diversity between swab and OmniGene samples were driven more by sample collection methods than transport times. Several clinically relevant taxa were shown to be affected by sample collection method and/or transport time. These results align and contribute to an established body of research highlighting some potential sources of methodological bias in 16S rRNA gene amplicon sequencing microbiome data. These data also illuminate potential limitations when comparing microbiome data derived from studies with different sampling and processing methodologies. There is a clear need for standardization of microbiome methods and increased efforts to standardize the use of publicly available data and to develop tools using artificial intelligence to correct for known biases.

## Supporting information

S1 Fig Alpha diversity rarefaction curves. Alpha diversity rarefaction curves for A) observed features and B) Shannon diversity up to 12,000 (12K) reads per sample. Rarefaction level for analyses performed in the current study was 10,000 (10K) reads per sample.
(TIF)

S2 Fig Genus-level taxonomic associations with transport time by sample collection method in nine genera with highest relative abundance that were significantly different based on sampling method. Scatter plot with line of best fit over transport time colored by sample collection method. *R* and *p*-values from Spearman correlation.
(TIF)

S3 Fig Weighted UniFrac biplots for swab samples by transport time. A) Phylum level. B) Class level. Taxa are depicted in red and the transport time experimental variable is depicted in black. Longer arrows with the same trajectory represent stronger associations. Arrows in opposite directions depict negative relationships.
(TIF)

S1 Table Differentially abundant phylum-, class-, and genus-level taxa between sample collection method. Footnote: Taxa with mean relative abundance over 5% denoted with *. Absolute value of log fold change is provided in table.
(XLSX)

S2 Table Differentially abundant taxa between sample collection method in subset non-shipped samples at the phylum-, class-, and genus-levels. Footnote: Taxa with mean relative abundance over 5% denoted with *. Absolute value of log fold change is provided in table.
(XLSX)
