# Supplementary figures and images for: Stabilized and unstabilized sampling methods result in differential fecal 16S rRNA microbial sequencing results
Source: PLoS One. 2025 Aug 13;20(8):e0324351. doi: 10.1371/journal.pone.0324351 (PMC12349053; doi:10.1371/journal.pone.0324351)

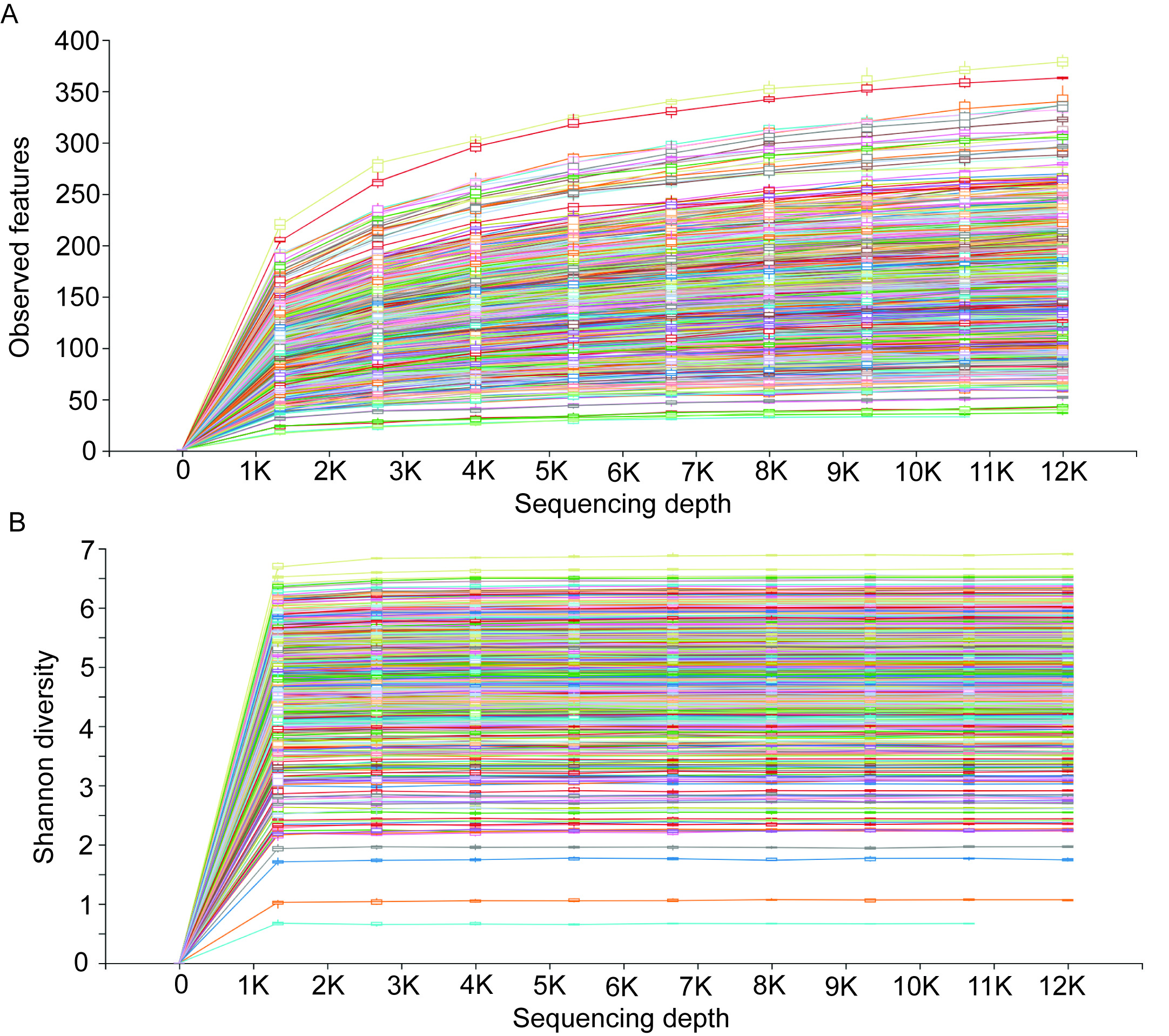

Supplement: S1 Fig — Alpha diversity rarefaction curves for A) observed features and B) Shannon diversity up to 12,000 (12K) reads per sample. Rarefaction level for analyses performed in the current study was 10,000 (10K) reads per sample. (TIF) [file pone.0324351.s001.tif]

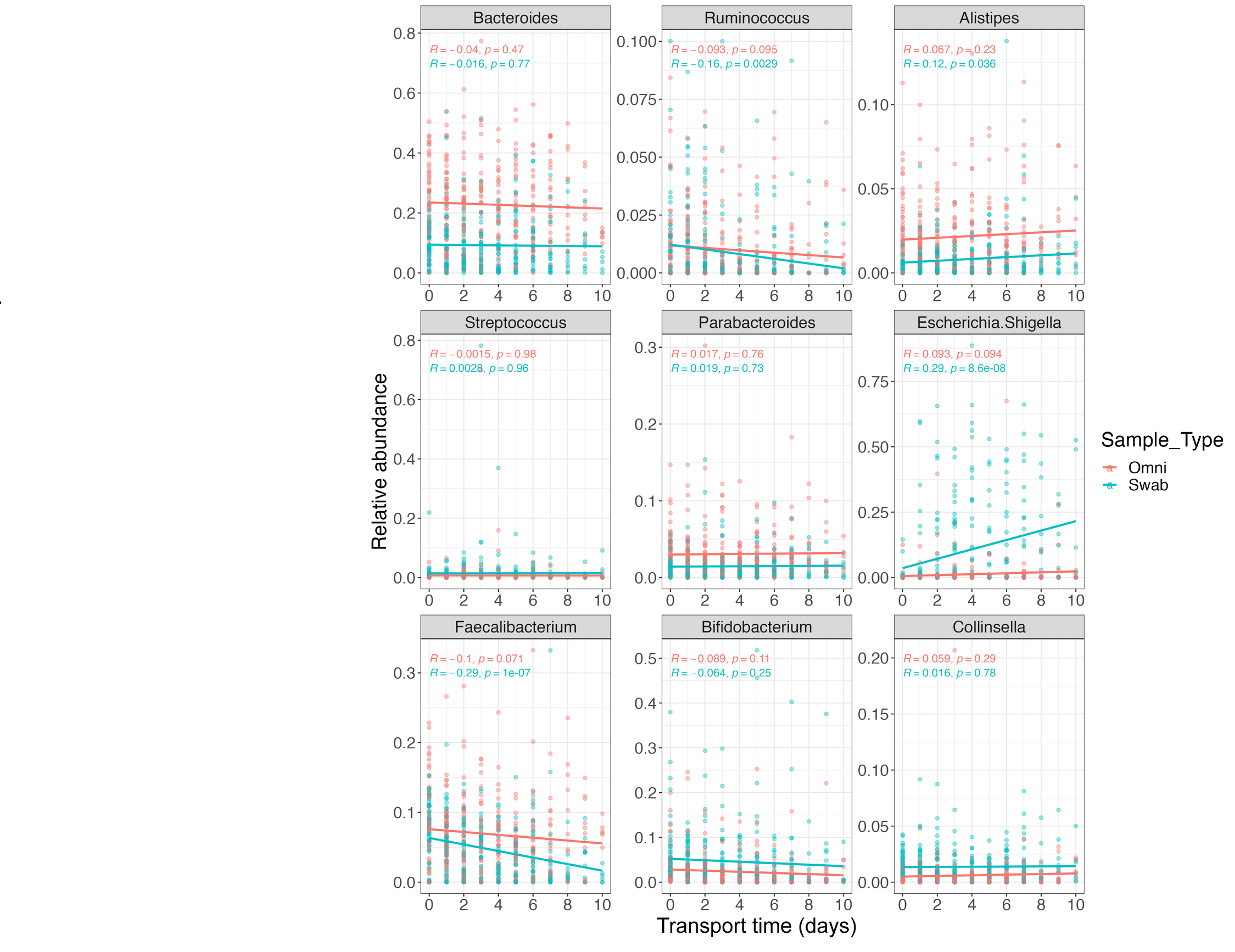

Supplement: S2 Fig — Scatter plot with line of best fit over transport time colored by sample collection method. R and p-values from Spearman correlation. (TIF) [file pone.0324351.s002.tif]

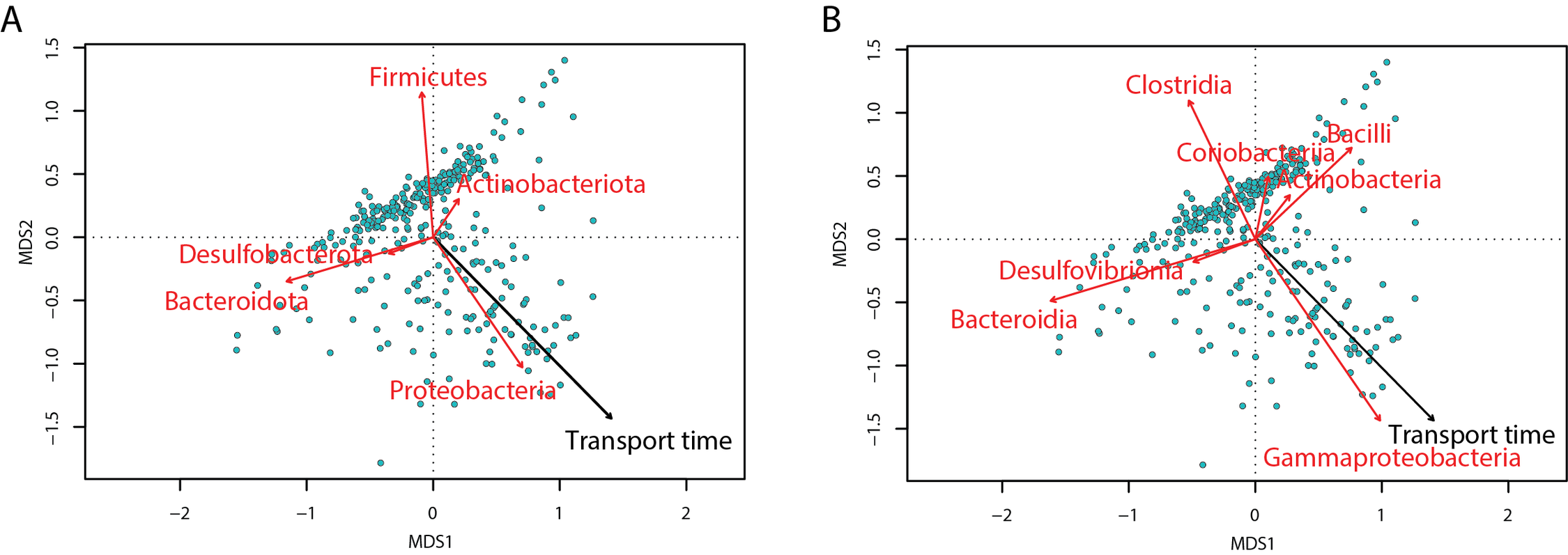

Supplement: S3 Fig — A) Phylum level. B) Class level. Taxa are depicted in red and the transport time experimental variable is depicted in black. Longer arrows with the same trajectory represent stronger associations. Arrows in opposite directions depict negative relationships. (TIF) [file pone.0324351.s003.tif]
